# The impact of cytomegalovirus coinfection on tuberculosis in the mouse model

**DOI:** 10.1186/s12879-026-12753-5

**Published:** 2026-03-04

**Authors:** Jaqueline Marschner, Sebastian Marwitz, Lars Eggers, Linda von Borstel, Lara Buer, David Hertz, Torsten Goldmann, Eleonore Ostermann, Wolfram Brune, Bianca E. Schneider

**Affiliations:** 1 Host Determinants in Lung Infections, Research Center Borstel, Leibniz Lung Center, Borstel, Germany; 2 Histology, Research Center Borstel, Leibniz Lung Center, Borstel, Germany; 3 Airway Research Center North, Member of the German Center for Lung Research (DZL), Großhansdorf, Germany; 4 Virus-Host-Interaction, Leibniz Institute of Virology, Hamburg, Germany

**Keywords:** Cytomegalovirus, Mycobacterium tuberculosis, Coinfection, Mouse model

## Abstract

**Background:**

Tuberculosis (TB) remains a major global health challenge, with 10.6 million new cases and 1.3 million deaths annually. HIV coinfection is a well-known risk factor in high-burden regions, but emerging evidence suggests that other viral infections, including cytomegalovirus (CMV), may also impact TB pathogenesis. CMV is a ubiquitous herpesvirus capable of lifelong persistence, and its potential role in modulating TB progression remains unclear.

**Methods:**

In this study, we established a murine coinfection model in C57BL/6 mice to investigate the effects of latent murine CMV (MCMV) on Mycobacterium tuberculosis (Mtb) infection. Mice were infected with either Mtb alone or with both Mtb and MCMV. Disease progression, mortality, bacterial load, viral clearance, and immune responses were assessed.

**Results:**

Contrary to our hypothesis, latent MCMV infection improved disease outcomes and reduced mortality following Mtb infection. This effect was independent of bacterial control or viral clearance. Instead, coinfected mice exhibited a distinct immunological environment in the lungs, with elevated levels of inflammatory mediators (IL-1α, IL-1β, TNF, IFN-γ, CXCL9, CXCL10, CCL2) and altered immune cell composition. Lesions in the lungs of coinfected mice were organized differently compared to those infected with Mtb alone. Notably, introducing MCMV during an established Mtb infection led to enhanced disease progression, highlighting the importance of the timing and sequence of infections.

**Conclusion:**

Our findings demonstrate for the first time that latent MCMV infection can improve disease outcomes in Mtb-infected mice, suggesting that viral latency may modulate the immune response in a way that enhances host defense against tuberculosis.

**Supplementary Information:**

The online version contains supplementary material available at 10.1186/s12879-026-12753-5.

## Background

Tuberculosis (TB) is the most prevalent bacterial infectious disease in humans and continues to be a major cause of morbidity and mortality worldwide [1]. The causative agent, *Mycobacterium tuberculosis* (*Mtb*), is carried by an estimated 2–3 billion people globally. In most cases it lies dormant and the immune system is able to prevent it from spreading in the body and causing symptoms of disease. However, the immune system fails to achieve sterile eradication of the tubercle bacillus, and 5–10% of infected people will develop active TB during their lifetime. The enormous reservoir of latent TB patients constantly leads to new active TB cases and transmission of the disease, thus perpetuating the epidemic [2]. Several risk factors for progression to active TB disease have been identified, of which coinfection with human immunodeficiency virus (HIV) is the most important one [3]. More recently, viral infections other than HIV have been suggested to play a role in the etiology of TB [4–6]. Epidemiological evidence supports an association between human cytomegalovirus (HCMV) and TB disease progression [7].

HCMV is a ubiquitous herpesvirus and establishes lifelong persistence in the host after primary infection which usually is oligosymptomatic in immunocompetent individuals [8]. The virus establishes latency in progenitor cells of the myeloid lineage and diverse populations of tissue stromal cells [9]. Reactivation may occur periodically but is generally well controlled by T-cell responses in healthy individuals and does not result in clinical presentation. However, lifelong viral carriage has a potential impact on the host immune response and may alter its efficacy towards infectious and non-infectious insult [10]. Several longitudinal and population cohort studies have indicated that being seropositive for HCMV was associated with the onset of vascular disease, various chronic inflammatory disorders, and specific types of human malignancies, diminished response to vaccinations, and increased susceptibility to infections [11–13]. As such, HCMV prevalence was higher among TB patients compared to healthy controls, and higher HCMV exposure was associated with active TB disease and lower levels of anti-mycobacterial antibodies [14–16]. Moreover, HCMV infection was shown to be a risk factor for TB disease in infants [17]. Given the epidemiological data, it is difficult to determine whether this association is causal or coincidental. Therefore, we established a new mouse model of CMV–*Mtb* coinfection to investigate whether CMV affects TB outcome. Surprisingly, latent CMV reduced disease severity and improved survival, while coinfection during established *Mtb* infection worsened outcomes, highlighting the importance of infection timing. These effects were linked to altered inflammation and immune cell recruitment rather than pathogen load. Our study provides a framework for exploring the mechanisms underlying CMV–*Mtb* interactions and their clinical implications.

## Materials and methods

### Ethics statement and mice

Animal experiments were in accordance with the German Animal Protection Law and approved by the Ethics Committee for Animal Experiments of the Ministry of Agriculture, Rural Areas, European Affairs and Consumer Protection of the State of Schleswig-Holstein (approval number 85 − 9/20). All mice used were purchased from Janvier Labs and maintained under specific barrier conditions in the BSL-3 facility at the Research Center Borstel. Female C57BL/6 mice aged between 10 and 13 weeks were used.

### Clinical score

A clinical score was used to assess severity of disease and disease progression. The clinical scoring followed predefined, objective criteria, and the experimenter was blinded to treatment groups during the scoring process to minimize bias. Animals were scored in terms of general behavior, activity, feeding habits, and weight gain or loss. Each of the criteria is assigned score points from 1 to 5 with 1 being the best and 5 the worst. The mean of the score points represents the overall score for an animal. Animals with severe symptoms (reaching a clinical score of ≥ 3.5) were euthanized to avoid unnecessary suffering, and the time-point was recorded as the end point of survival for that individual mouse.

### Cells

M2-10B4 (CRL-1972) cells were obtained from the American Type Culture Collection. Murine 10.1 fibroblasts are spontaneously immortalized mouse embryonic fibroblasts (MEFs) from BALB/c mice obtained from Thomas Shenk (Princeton University, USA) [18]. M2-10B4 and 10.1 cells were cultured in complete Dulbecco’s modified Eagle medium (DMEM) supplemented with 10% fetal calf serum (FCS), 100 U/ml penicillin, and 100 µg/mL streptomycin. Bone marrow-derived macrophages (BMDM) were generated from bone marrow isolated from female C57BL/6 mice provided by the animal facility of Research Center Borstel. After euthanasia, mice were disinfected with 70% ethanol and tibia and femur were removed, cleaned of tissue, and the marrow was flushed into a 50 mL conical tube using a 23-gauge needle with 20 mL of cold BMDM medium (DMEM supplemented with 10% (v/v) FCS (heat inactivated), 20% (v/v) L929 cell culture supernatant (filtered), 5% (v/v) horse serum, 2 mM L-Glutamine, 1 mM Sodium pyruvate and 10 mM HEPES) per leg. Cells were centrifuged at 250 x *g* for 5 min at 4 °C, and the supernatant was discarded. For BMDM differentiation, cells were resuspended in BMDM medium and incubated at 37 °C and 5% CO_2_ for 6–7 days, with 5 mL additional medium added after 3 days. After differentiation, BMDMs were harvested using cold DPBS and a sterile rubber policeman, and resuspended in BMDM medium for further incubation and experiments.

### MCMV infection

The WT-MCMV virus used in this study was based on the pSM3fr-MCK-2-fl BAC [19] and modified to delete m157 as described [20] in order to prevent Ly49H-mediated NK cell activation in C57BL/6 mice [21]. Infectious virus was reconstituted from BAC DNA by transfection of 10.1 fibroblasts. For stock production, virus was propagated in 10.1 fibroblasts and purified from supernatant by ultracentrifugation through a sucrose cushion as described [22]. Virus stocks were titrated on M2-10B4 cells by standard plaque assay (24). For in vivo infection, mice were anaesthetised (12.5% ketamine, 1,25% xylazine in 1x PBS; 200 µL / 20 g body weight) and inoculated intranasally (i.n.) with 20 µL of 2*10^5^ PFU of MCMV-Δm157. The inoculum was slowly and evenly applied to one nostril. For in vitro infection, cells were infected at the desired multiplicity of infection (MOI) with MCMV-Δm157. After 4 h, the medium was changed and the cells were supplied with fresh medium and further incubated for the indicated time.

### Mtb infection

*Mtb* H37Rv was grown in Middlebrook 7H9 broth (BD Biosciences) supplemented with 10% v/v OADC (Oleic acid, Albumin, Dextrose, Catalase) enrichment medium (BD Bioscience), 0.2% v/v glycerol and 0,05% v/v Tween 80 to logarithmic growth phase (OD600 0.2–0.4) and bacterial aliquots were frozen at − 80 °C. Viable cell number in thawed aliquots were determined by plating serial dilutions onto Middlebrook 7H10 agar plates supplemented with 10% v/v heat-inactivated bovine serum followed by incubation at 37 °C for 3–4 weeks. For infection of experimental animals, *Mtb* stocks were diluted in sterile distilled water at a concentration providing an uptake of 100 viable bacilli per lung. Infection was performed via the respiratory route by using a Glas-Col aerosol chamber as described previously [23].

### Determination of colony forming units (CFU)

Bacterial loads were evaluated at different time points after aerosol infection by mechanically disrupting the lungs, mediastinal lymph nodes, and spleens in PBS. Tenfold serial dilutions of the organ homogenates in sterile water, 1% v/v Tween 80, 1% w/v albumin were plated onto Middlebrook 7H11 agar plates supplemented with 10% v/v heat-inactivated bovine serum. The plates were incubated at 37 °C, and colonies were enumerated after 3–4 weeks.

### Plaque assay

Viral titers in cell culture supernatants were determined by plaque assay. A total of 4 × 10^4^ M2-10B4 cells were seeded in 48-well plates one day prior to infection. On the following day, serial log_10_ dilutions (ranging from 10^− 1^ to 10^− 4^) were prepared in 1 mL of 3% FCS growth medium for each sample. Organ homogenates were titrated in duplicates, cell supernatant in 4 independent serial dilutions. From each dilution, 100 µL was added to the respective wells. After a 3-hour incubation period at 37 °C, 300 µL of methylcellulose (viscosity: 4,000 cP, 2.5% in water) was added to each well. Five to six days post-infection, plaques were counted, and the viral titer was calculated as described by Zurbach et al. [24].

### Absolute quantitative PCR

To determine viral genome copies in different organs, total DNA was extracted from organ homogenates diluted 1:3 in TRIZOL LS following the protocol of the van de Rijn Laboratory in Stanford, USA (https://med.stanford.edu/vanderijn/Protocols.html). The DNA concentration and purity were determined using a NanoDrop 1000 (Thermo Fisher Scientific Inc.). qPCR targeting the MCMV IE1 gene was performed using the QuantStudio 3 Real-Time PCR system. Serial dilutions of pcDNA 3-encoding MCMV IE1 or mouse beta-actin were used as standard. Each reaction consisted of ~ 100 ng DNA, 5 µL of PowerTrack SYBR Green Master Mix (Thermo Fisher Scientific Inc.), 0.5 µL of each forward and reverse primer (10 µM), and nuclease-free water to a final volume of 10 µL. The thermal cycling conditions were as follows: 95 °C for 15 s, annealing and elongation at 60 °C for 60 s, followed by a dissociation stage. Analysis of the absolute quantification was performed using the QuantStudio Design and Analysis Software (version 2.7.0, Thermo Fisher Scientific Inc.). All quantifications were normalized to the beta-actin copies number. The following primers were used: beta-actin forward AGAGGGAAATCGTGCGTGAC, reverse CAATAGTGATGACCTGGCCGT; IE1 forward ACTAGATGAGCGTGCCGCAT, reverse TCCCCAGGCAATGAACAATC. Ten MCMV genome copies were defined as the lowest concentration of viral genome that can be reliably detected and measured by the qPCR performed in this study.

### Multiplex immunoassay

The concentration of cytokines and chemokines in lung homogenates was quantified by LEGENDplex (Mouse inflammation panel, Biolegend) according to the manufacturer’s instructions. Data were acquired on a MACSQuant 10 flow cytometer (Miltenyi) and analyzed with the LEGENDplex software (Biolegend).

### TNF quantification

TNF levels in cell supernatants were quantified using the TNF ELISA MAX kit (BioLegend), following the manufacturer’s protocol. Absorbance was measured at 450 nm using a microplate reader. TNF concentrations were calculated based on a standard curve generated from known concentrations of TNF standards.

### Histology

Superior lung lobes from infected mice were fixed with 4% w/v paraformaldehyde for 24 h, embedded in paraffin, and sectioned (4 μm). Sections were stained with hematoxylin and eosin (H&E) to assess overall tissue pathology. Slides were imaged with a PhenoImager HT instrument (Akoya Biosciences, Marlborough, USA). The quantitative analysis of inflammatory area was conducted by determination of whole lung area and inflammatory area for H&E-stained sections using the software QuPath [25].

### Multiplex-immunofluorescence (mIF) staining

4 μm sections from formalin-fixed paraffin embedded tissue were mounted on SuperFrost+ microscope slides, deparaffinized and re-hydrated by 2x xylene (10 min.), 2 × 100% EtOH (2 min.), 1 × 96% EtOH (2 min.), 1 × 90% EtOH (2 min.), 1 × 80% EtOH (2 min.), 1 × 70% EtOH (2 min.) and Millipore H_2_O (2 min.). Tissue area was encircled with a Pap pen and the slides subsequently transferred to 1x TBST (50 mM Tris, pH 7.6, 0.05% Tween-20). The Opal 3-Plex Anti-Rb Manual Detection Kit (Akoya Biosciences, Marlborough, USA, NEL840001KT) was used with additional fluorophores for all mIF stainings. The general mIF staining was conducted as described elsewhere [26]. Overall, mIF staining consisted of several, subsequent cycles of IF staining targeting one antigen per time: Before antigen detection, endogenous peroxidases were blocked once by incubation for 10 min at RT in 3% H_2_O_2_ followed by 3 × 2 washing with 1 x TBST. One IF cycle consisted of 10 min protein blocking using antibody diluent (Akoya Biosciences, Marlborough, USA) followed by 3 × 2 min washing with 1x TBST. Primary antibodies were diluted using antibody diluent (Akoya Biosciences, Marlborough, USA) and incubated for 45 min at RT followed by 3 × 3 min washing with 1 x TBST. Primary antibodies were detected by incubation for 10 min with HRP-Polymer (Akoya Biosciences, Marlborough, USA) and subsequent TBST washing for 3 × 3 min. Signals were visualized by OPAL-TSA reaction for 10 min at RT and stopped by washing 3 x for 3 min with 1x TBST. An antigen-retrieval (AR) step by heating in citric acid buffer pH 6 (Akoya Biosciences, Marlborough, USA) for 1 min at 1000 W followed by simmering for 10 min at 100 W was used to remove primary antibodies and HRP polymer before entering the next staining cycle. Finally, nuclei were stained using spectral DAPI (Akoya Biosciences, Marlborough, USA) and slides were mounted with cover slips using ProLong Gold (Invitrogen, Carlsbad, USA). A detailed pipetting protocol for the applied mIF panel, used primary antibodies, dilutions and fluorophores can be found in Supplementary Table S1.

### mIF imaging & digital image analyses

Whole-slide imaging (WSI) was performed using a PhenoImager HT multi-spectral slide scanner (Akoya Biosciences, Marlborough, USA) at 20 x magnification (0.5 μm/pixel) with exposure times held constant for all analyzed samples (DAPI: 1.1 ms, Opal 690: 30 ms, Opal 520: 35 ms, Opal 570: 43.12 ms, Opal 780: 25 ms). Phenochart software 1.1.0 (Akoya Biosciences, Marlborough, USA) was used to select regions of interest for generation of image analysis algorithms as well as batch analyses across all tissues. For this, an analysis algorithm was built using InForm software 2.6.0 (Akoya Biosciences, Marlborough, USA) with two representative regions from each tissue. Due to the heterogeneous nature of lung involvement in *Mtb* infection, analysis was restricted to representative regions containing lesions to accurately quantify local immune cell recruitment. The image analysis algorithm consisted of several steps including spectral unmixing based on proprietary spectral libraries of designated fluorochromes as supplied by Akoya Biosciences, Marlborough, USA, pixel-based tissue segmentation, cell segmentation, and cell classification. Tissue segmentation was tested on randomly selected WSIs and retrained on areas where initial tissue segmentation failed to correctly identify designated areas. Representative regions from WSIs were imported into InForm software, spectrally unmixed, and forwarded to user-guided, machine-learning tissue segmentation based on manual annotation for areas consisting either of empty glass/no tissue, B cell enriched tissue, and tissue without B cell enrichment. Once tissue segmentation reached a satisfactory level, individual cells were segmented based on their DAPI signal and accessory information from antibody staining. A cell classification algorithm was trained to identify each antigen in a binary approach (antigen positive vs. negative).

Two representative regions from each WSI were forwarded to batch analysis. All resulting images were individually reviewed, and areas containing imaging artifacts (dust, fibers, tissue folds, out-of-focus regions) as well as pleura with excess autofluorescence were removed by manually drawing regions of disinterest. Samples with insufficient tissue segmentation results were excluded from downstream analyses. Resulting cell segmentation files of 128 images from 64 WSIs were merged and consolidated into a single data file using the R package phenoptr reports 0.3.3. Phenoptr reports was further used to determine the cell density (cells/mm²) of two images/WSI for selected combinations of detected antigens across the complete tissue of each animal. The slides were analyzed in a blinded manner.

### Statistical analysis

All data were analyzed and graphs generated using GraphPad Prism 10 (GraphPad Software, La Jolla, USA). Statistical tests are indicated in the individual figure legends. An ordinary 2-way ANOVA (comparison of 3 or more groups) was performed with a Tukey’s (for comparisons involving three or more groups to assess all possible pairwise differences) or Sidak’s (for comparisons involving only two groups to control for multiple testing with fewer comparisons) multiple comparisons test (95% confidence interval). The survival curve analysis was performed using the log rank (Mantel-Cox) test. Unless otherwise defined, the differences were considered significant if p-values were ≤ 0.05 and are specified and marked with stars as the following: * *p* ≤ 0.05; ** *p* ≤ 0.01; *** *p* ≤ 0.001; **** *p* ≤ 0.0001. Unless otherwise stated, data are shown as means with the corresponding standard deviation (SD).

## Results

### Latent MCMV infection reduces disease progression and mortality following *Mtb* infection

**Fig. 1:**
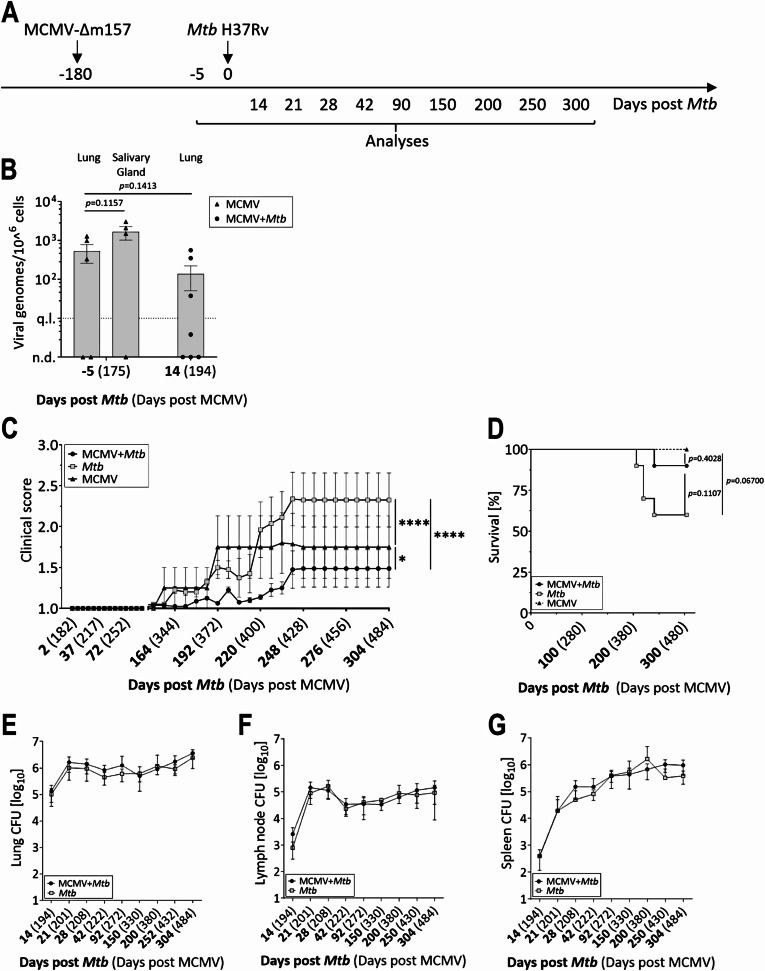
Latent MCMV infection modulates disease progression and survival in coinfected mice. (**A**) Timeline of experimental setup. C57BL/6 mice were infected i.n. with 2x105 PFU MCMV-Δm157 and 180 days later with a low dose of Mtb H37Rv. (**B**) Quantification of viral genomes by qPCR in lung and salivary gland tissue collected 5 days before and 14 days after Mtb infection; normalized to beta-actin. The black dotted line represents the qPCR quantification limit (n=4-7, data pooled from two independent experiments; n.d. = not detectable; q.l. = quantification limit; each data point represents one mouse.). (**C**) Clinical score and (**D**) survival were monitored until day 304 after Mtb infection (n=7-10 per group, data from one experiment). At indicated time points, bacterial burden in lung (**E**), lymph node (**F**) and spleen (**G**) were determined (n=6-9, data pooled from two independent experiments). *p ≤ 0.05; **p ≤ 0.01, ***p ≤ 0.001, ****p ≤ 0.0001 determined by (**B**) Unpaired t-test, (**C**) 2-way ANOVA followed by Tukey’s multiple-comparison test, (**D**) log-rank (Mantel-Cox) test and (**E**-**G**) 2-way ANOVA followed Sidaks multiple comparison test

To assess the impact of MCMV infection on the outcome of *Mtb* infection, we established a coinfection model in C57BL/6 mice. The B6 inbred strain is the most widely used inbred mouse strain in TB research. It is permissive to infection with virulent *Mtb* but efficiently controls bacterial replication due to the development of T cell-mediated immunity [27]. This mouse strain is however MCMV-resistant due to NK cell activation via the viral glycoprotein m157 [28]. This resistance can be prevented by using a Δm157 MCMV unable to bind to the NK cell receptor Ly49H [20]. It has been demonstrated before that MCMV establishes latency in the lungs when administered via the mucosal route, one important natural route of infection [29]. Therefore, female C57BL/6 mice were infected i.n. with 2*10^5^ PFU of MCMV-Δm157 (Fig. 1A) and 6 months later, latent viral genome load was determined by qPCR. Low levels of viral genome were detected in most samples 175 days after MCMV infection, with more copies being detected in the salivary gland, which is the primary site of MCMV latency [30] compared to the lung (Fig. 1B).

Next, mice latently infected with MCMV were aerosol-infected with a low dose of *Mtb* H37Rv (Fig. 1A). First, we investigated whether *Mtb* infection triggered reactivation of latent MCMV. However, the levels of the viral genome detected 14 days after coinfection remained comparable to those observed prior to coinfection (Fig. 1B). Then, we monitored TB disease progression in the presence and absence of the virus. The clinical score began to increase 150 days post-*Mtb* infection in both groups, while mice infected with MCMV alone showed hardly any symptoms throughout the observation period, resulting in a consistently low clinical score (Fig. 1C). Unexpectedly, the clinical score was highest in the group of mice infected with *Mtb* alone and significantly lower in the presence of MCMV. Consequently, 4 out of 10 *Mtb*-only-infected animals had to be euthanized upon reaching the humane endpoint, compared to only 1 out of 10 coinfected mice (Fig. 1D), indicating that latent MCMV infection ameliorated disease progression.

We next examined whether the improved condition of coinfected mice was due to an enhanced ability to control bacterial loads. Organs of interest were collected throughout the observation period to assess the impact of the underlying viral infection on *Mtb* control. However, the bacterial loads in lung, spleen and mediastinal lymph node were comparable between *Mtb*-only and coinfected mice (Fig. 1E), indicating that differences in disease outcome were independent from the control of bacterial replication.

Together, these data demonstrate that latent MCMV infection has a beneficial effect on coinfected mice. Contrary to our hypothesis, these mice exhibited an improved disease outcome compared to those infected with *Mtb* alone.

### Coinfected animals exhibit elevated levels of inflammatory cytokines and chemokines in the lungs

**Fig. 2:**
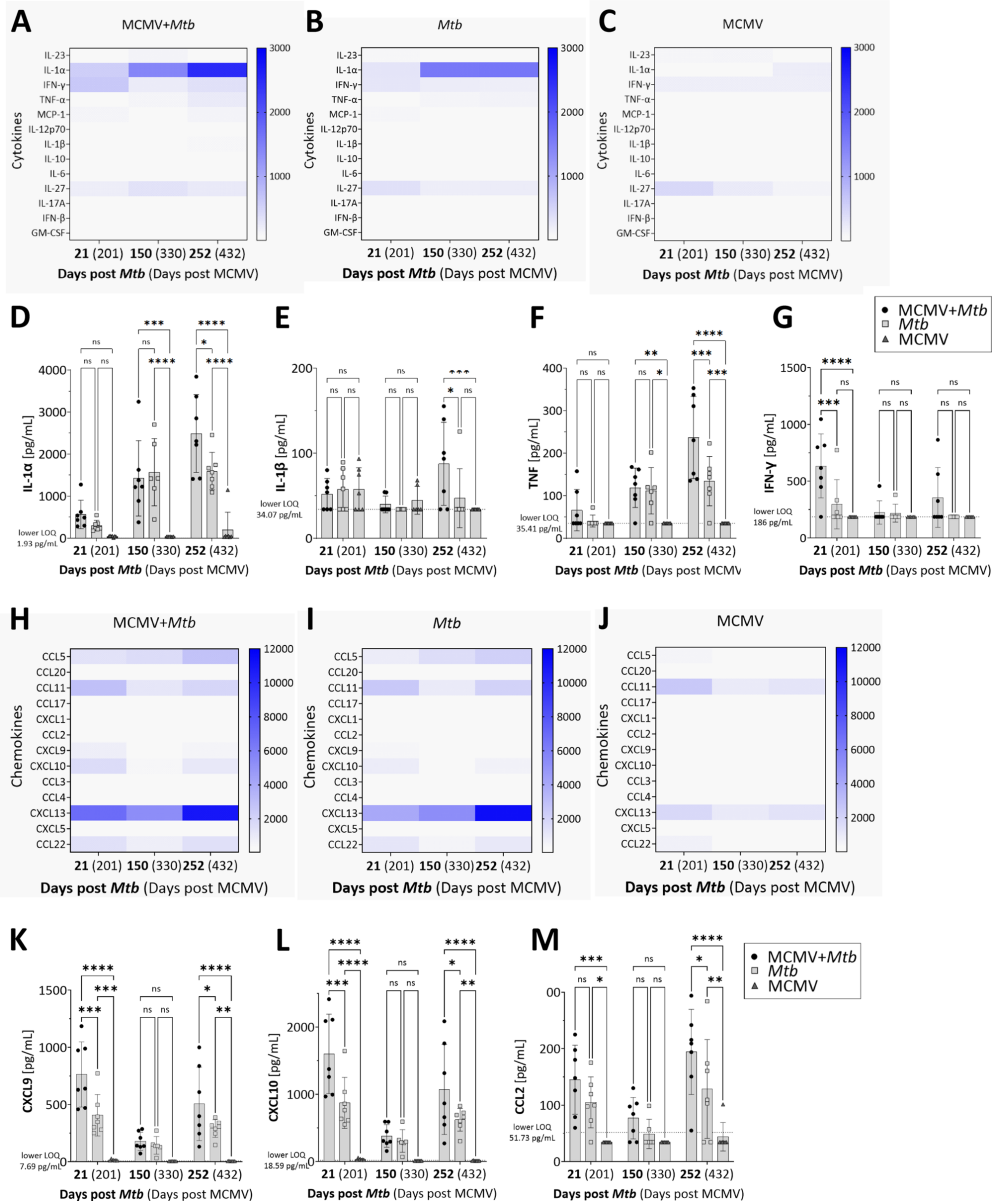
Altered pulmonary cytokine and chemokine responses in coinfected mice. Heat maps of cytokine (**A**-**C**) and chemokine levels (**H**-**J**) in lung homogenates. Concentration of selected cytokines (**D**-**G**) and chemokines (**K**-**M**) at representative time points (*n* = 6–9, data pooled from two independent experiments). LOQ = Limit of quantification. Each data point represents one mouse. *p ≤ 0.05; **p ≤ 0.01, ***p ≤ 0.001, ****p ≤ 0.0001 determined by 2-way ANOVA followed by Tukey’s multiple-comparison test

To examine the immunological environment in the *Mtb*-infected lungs that accompanied improved disease outcomes in the presence of latent MCMV, we quantified the levels of inflammatory cytokines and chemokines known to play a role in *Mtb* infection. Among the various cytokines measured, IL-1α was produced at the highest level in both co- and *Mtb*-only infected animals (Fig. 2A, B). Together with IL-1β and TNF its concentration increased over time, reaching significantly higher levels in coinfected compared to *Mtb*-infected mice in the late infection phase (day 252 post *Mtb* infection; Fig. 2D-F) when differences in clinical score were most apparent (Fig. 1C). In contrast, IFN-γ levels peaked early at day 21 after *Mtb* infection, with significantly higher levels also observed in coinfected animals compared to those infected with *Mtb* alone (Fig. 2G). Moreover, several chemokines were induced in coinfected and *Mtb*-only infected animals (Fig. 2H-J). Of those, CXCL9, CXCL10, and CCL2 (Fig. 2K-M) were significantly higher in coinfected mice at an early (day 21) and late time point (day 252). These findings highlight an altered cytokine and chemokine profile in the lungs of coinfected mice, particularly during the late phase of infection, associated with improved clinical outcomes.

### Histopathological changes in lung tissue in the presence and absence of latent MCMV

**Fig. 3:**
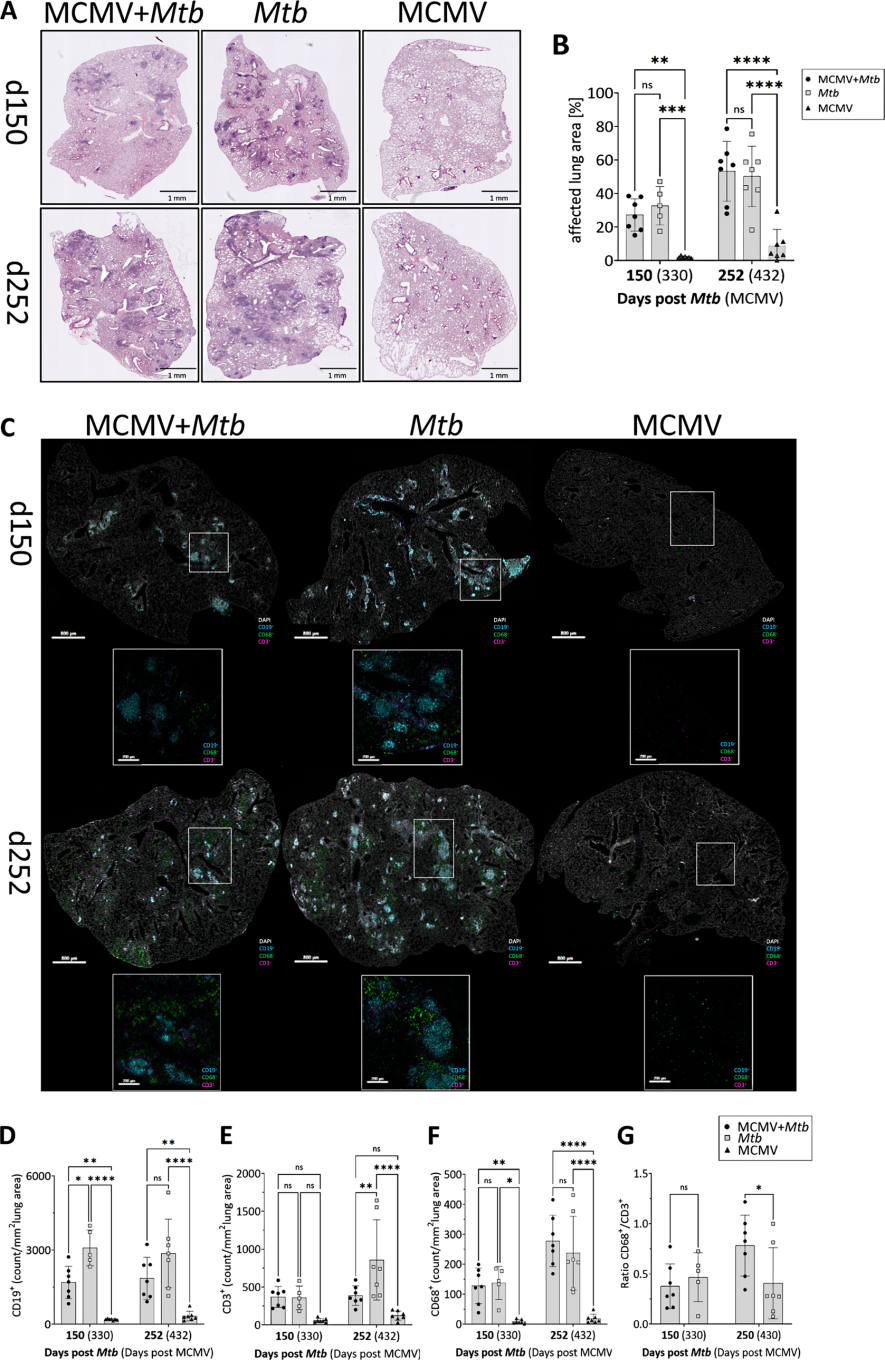
MCMV coinfection changes cellular infiltration into the Mtb-infected lung. Lungs were collected at day 150 (equivalent to 330 days post MCMV) and 252 days post Mtb infection (432 days post MCMV), and PFA-fixed, paraffin embedded tissue sections were stained with H&E (**A**). Representative micrographs from one mouse per group are shown. Bar = 1 mm. (**B**) Quantitative analysis of the area of lung inflammation as shown in (**A**). n=5-7, data pooled from two independent experiments. (**C**) Representative micrographs from lungs of one mouse per group 150 days post Mtb (equivalent to 330 days post MCMV) and 252 days post Mtb (432 days post MCMV), stained with antibodies to detect B cells (CD19+), T cells (CD3+), and macrophages (CD68+); Bar = 800 µm; micrographs in detail bar = 200 µm. (**D**-**F**) Quantitative analysis of immune cell densities based on the mean of two images/WSI as shown in (**C**). n=5-7, data pooled from two independent experiments. (**G**) Ratio of CD68+ macrophages to CD3+ T cells. *p ≤ 0.05; **p ≤ 0.01, ***p ≤ 0.001, ****p ≤ 0.0001 determined by (**B**; **D**-**G**) 2-way ANOVA followed by Tukey’s multiple-comparison test

To evaluate the impact of latent MCMV coinfection on pulmonary pathology, one lung lobe was processed for histological analysis. Overall pathological changes were assessed using Hematoxylin & Eosin (H&E) staining. Inflammatory regions in the lungs of *Mtb*-only and coinfected mice exhibited intense violet-blue staining, indicative of dense lymphocyte and macrophage infiltration (Fig. 3A). Interestingly, while there was no significant difference in the overall lung region affected between mice infected solely with *Mtb* and those coinfected at both time points investigated (Fig. 3B), visual assessment indicated notable differences in cellular composition, especially on day 150 post-*Mtb* infection (Fig. 3A). Therefore, we performed multiplex immunofluorescence (mIF) to identify CD19^+^ B cells, CD3^+^ T cells, and CD68^+^ monocytes and macrophages (Fig. 3C). Quantification revealed a high abundance of CD19^+^ B cells during the mid and late phases of *Mtb* infection (days 150 and 252), which was lower in the presence of MCMV, reaching significance at day 150 (Fig. 3D). Similarly, the abundance of CD3^+^ T cells was highest in the lungs of *Mtb*-only infected mice, particularly on day 252 (Fig. 3E). In contrast, no significant differences were observed in the abundance of CD68^+^ macrophages between the two groups, although there was a trend toward more CD68^+^ cells in the lungs of coinfected mice at day 252 (Fig. 3F). Consequently, the ratio of CD68^+^ macrophages to CD3^+^ T cells was significantly higher in the coinfected lungs (Fig. 3G). This observation is consistent with our findings of increased production of the monocyte-attracting chemokine CCL2 in coinfected mice (Fig. 2M). It also aligns with the elevated levels of IL-1α, IL-1β, and TNF, primarily produced by macrophages in response to *Mtb*.

In contrast to the *Mtb*-infected lungs, MCMV-only infected lungs (Fig. 3A) showed minimal pathological changes at mid-infection (day 150 post *Mtb*, which corresponds to 330 days post MCMV infection). However, 100 days later, we observed larger areas of consolidation (day 252), but the pathology was clearly distinct from the granulomatous lesions as observed in all *Mtb*-infected lungs (Fig. 3A).

### Macrophage responses to MCMV and *Mtb* coinfection

**Fig. 4:**
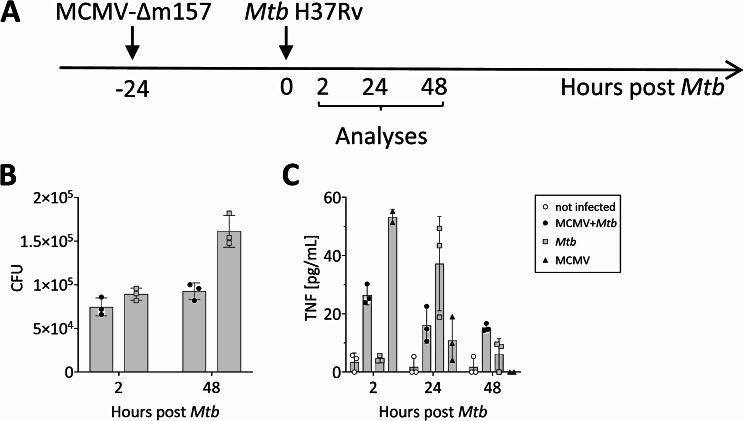
Impact of MCMV coinfection on macrophage activation and bacterial control. (**A**) Timeline. BMDMs isolated from C57BL/6 mice were infected with MCMV-Δm157-WT MOI 1 and one day later coinfected with an MOI of 0.3 *Mtb* H37Rv. (**B**) At the indicated time points, serial dilutions were plated onto Middlebrook 7H11 agar plates for CFU determination. (**C**) TNF production was determined in the cell supernatant at the indicated time points. Data from one experiment representative of two independent experiments are shown as mean + SEM from technical triplicates

Macrophages represent one of the most important target cells for both *Mtb* and MCMV [31–33]. Interestingly, it was previously shown that MCMV subverts macrophage identity and impairs their antimicrobial effector functions, including the ability to phagocytose particles and to produce TNF in response to LPS stimulation [31, 32, 34, 35]. To further explore how MCMV coinfection affects macrophage responses to *Mtb*, we infected BMDMs isolated from female C57BL/6 mice with MCMV-Δm157 (MOI 1) and coinfected them with *Mtb* H37Rv (MOI 0.3) the following day (Fig. 4A). Bacterial loads in both *Mtb*-only and coinfected BMDMs were comparable at 2 h post-infection, indicating that MCMV infection did not affect *Mtb* uptake by BMDMs. By 48 h post-infection, bacterial numbers increased in *Mtb*-only infected cells, whereas coinfected cells maintained stable bacterial loads (Fig. 4B), suggesting enhanced control in macrophages previously infected with MCMV. To evaluate macrophage activation, we measured TNF release. Immediately following *Mtb* infection (2 h), TNF levels in the supernatant of *Mtb* only-infected cells were comparable to those of uninfected cells. However, cells previously infected with MCMV released TNF into the supernatant (Fig. 4C). At 24 h, TNF levels had decreased in MCMV-infected cells but increased in *Mtb*-infected cells. By 48 h post-*Mtb* infection, TNF levels were low and comparable between coinfected and *Mtb*-only infected cells.

Together, these findings indicate that a prior infection with MCMV activates macrophages, as evidenced by the earlier release of TNF, and enhances their ability to control a subsequent *Mtb* infection.

### MCMV coinfection: beneficial only in latent stages

**Fig. 5:**
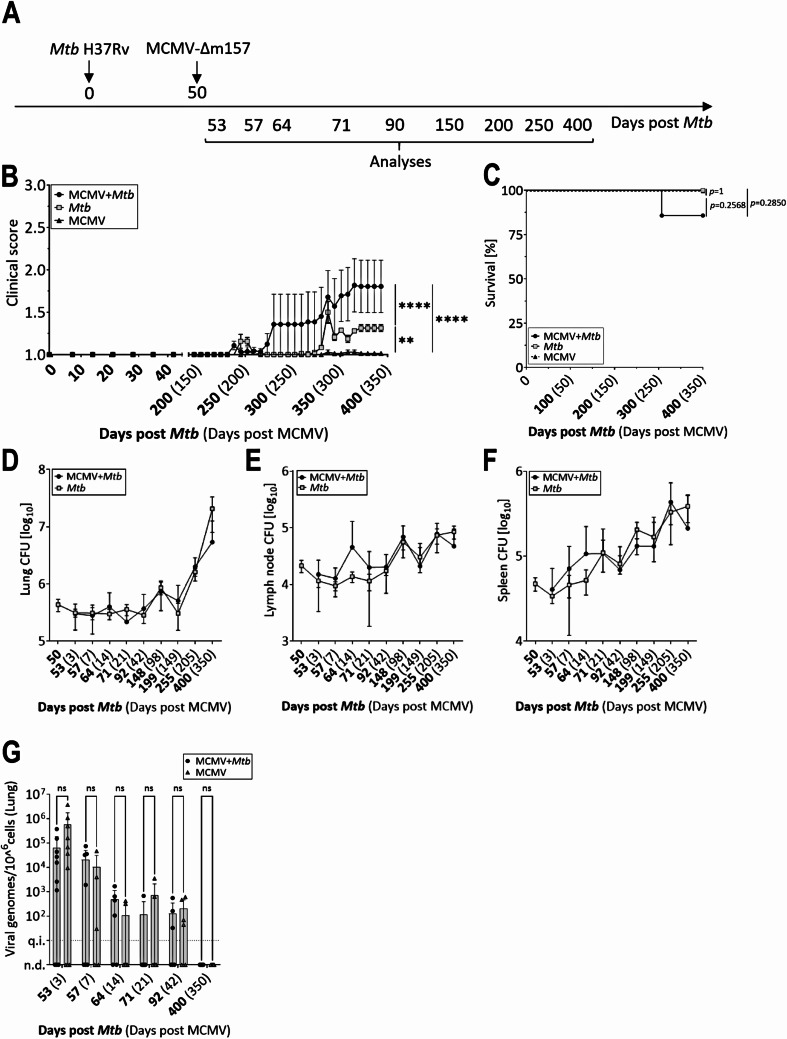
Timing of MCMV coinfection determines disease outcome in MCMV/Mtb-infected mice. (**A**) Timeline. C57BL/6 mice were infected with a low dose of *Mtb* H37Rv and 50 days later i.n. with 2 × 10^5^ PFU MCMV-Δm157. (**B**) Clinical score and (**C**) survival (*n* = 7–9 per group, data from one experiment). At indicated time points, bacterial burden in lung (**D**), lymph node (**E**) and spleen (**F**) were determined (*n* = 6–12 per group, data pooled from three independent experiments). **(G)** Quantification of viral genomes by qPCR in lung collected at the indicated time points (*n* = 5–10, data pooled from three independent experiments; n.d. = not detectable; q.l. = quantification limit). Each data point represents one mouse. *p ≤ 0.05; **p ≤ 0.01, ***p ≤ 0.001, ****p ≤ 0.0001 determined by **(B)** 2-way ANOVA followed by Tukey’s multiple-comparison test, **(C)** log-rank (Mantel-Cox) test, **(D**-**F)** 2-way ANOVA followed Sidaks multiple comparison test

It is known that the sequence and the timing of coinfections is important and can have a profound impact on the outcome of disease [36]. Having observed the positive impact of a preceding latent MCMV infection on disease progression during subsequent *Mtb* infection, we sought to investigate whether MCMV infection occurring only when chronic *Mtb* infection was already established would have a similar effect. To do so, mice were infected with a low dose *Mtb* H37Rv and, 50 days later - when TB takes a chronic but controlled course - administered MCMV-Δm157 i.n. (Fig. 5A). Again, disease progression was monitored and bacterial loads determined at the indicated time points. The frequent initial analyses were intended to evaluate the immediate effects of an acute MCMV infection on the control of *Mtb*, as well as to assess the potential impact of *Mtb* infection on viral clearance. Additionally, the impact of MCMV coinfection on the long-term control of *Mtb* was assessed through extended observation. While the MCMV-infected mice did not exhibit any signs of disease throughout the observation period, the clinical score of *Mtb*-infected mice began to increase around day 230. In contrast to our observations in mice latently infected with MCMV, the score of coinfected animals was significantly higher than that of animals infected with *Mtb* alone (Fig. 5B). Overall, the disease burden in these animals was moderate, and only one coinfected mouse had to be euthanized because it reached the humane endpoint before the end of the observation period (Fig. 5C).

Despite increased clinical symptoms, bacterial loads in the lungs, mediastinal lymph nodes, and spleen were comparable between *Mtb*-only and coinfected mice, suggesting the deterioration was unrelated to uncontrolled bacterial replication (Fig. 5D-F). Absolute quantification of MCMV genome showed slightly reduced viral loads in the lungs of coinfected mice on day 3, with greater reductions by day 7, but no significant differences at later time points (Fig. 5G). By day 350, no detectable virus remained in either group.

In conclusion, although preexisting latent MCMV had a beneficial effect, coinfection with MCMV during an already established *Mtb* infection exacerbated clinical symptoms, underscoring the critical role of timing and sequence in coinfection outcomes.

## Discussion

Growing epidemiological evidence indicates that HCMV infection is associated with an increased risk of progressing from latent to active TB [7, 14–17]. However, experimental insights into the precise immunological alterations induced by CMV coinfection and its impact on *Mtb* control remain limited. To address this gap, the present study investigated MCMV-*Mtb* coinfection in C57BL/6 mice, aiming to understand how MCMV coinfection influences the progression and outcome of *Mtb* infection under controlled experimental conditions. Surprisingly, our findings revealed that latent MCMV infection ameliorated disease progression and reduced mortality following *Mtb* infection. These results are consistent with previous murine studies showing that prior MCMV infection can enhance resistance to heterologous pathogens such as vaccinia, lymphocytic choriomeningitis, and Pichinde viruses [37, 38]. Notably, this protective effect was not restricted to secondary viral infections but also extended to bacterial pathogens. For example, latent MCMV infection provided substantial protection against *Listeria monocytogenes* and *Yersinia pesti*s [39]. Latency-induced protection was characterized by prolonged production of the pro-inflammatory cytokines IFN-γ and TNF, along with extensive activation of macrophages, which led to a notable decrease in bacterial load. By contrast, in our coinfection model, although IFN-γ and TNF were increased, improved disease outcomes occurred without reductions in bacterial load, suggesting that latent MCMV infection enhanced host tolerance to *Mtb* rather than directly reducing bacterial burden. Disease tolerance is a distinct facet of host defense, which mitigates the deleterious consequences of infection—such as tissue damage and immunopathology—thereby preserving host fitness despite persistent infection [40].

Consistent with this, the lungs of coinfected mice displayed distinct immunological profiles compared to those infected with *Mtb* alone. Despite elevated levels of the T cell–attracting chemokines CXCL9 and CXCL10, the abundance of CD3⁺ T cells did not increase; in fact, both CD3⁺ T cells and CD19⁺ B cells were significantly reduced. These observations suggest that latent MCMV infection may modulate the recruitment or maintenance of adaptive immune cells in the lung, potentially dampening excessive inflammatory responses that contribute to tissue damage. B and T cells are key components of TB granulomas, often organized within tertiary lymphoid structures that coordinate local immune responses. CD4⁺ T cells are essential for controlling *Mtb*, but excessive or dysregulated Th1 responses can lead to lung tissue damage [41, 42]. B cells similarly play a dual role in TB: they can be protective by supporting granuloma structure and immune coordination, yet pathogenic or regulatory when dysregulated, contributing to tissue damage or dampening bacterial clearance [26, 43, 44]. Interestingly, B cell deficient mice were shown to display lower levels of inflammation during chronic *Mtb* infection [45, 46]. Reduced inflammation was associated with decreased CD4^+^ T cell proliferation and a survival advantage relative to WT animals.

In contrast to B and T cells, the number of CD68⁺ macrophages was higher during the late stages of coinfection compared to *Mtb*-only infection, consistent with elevated CCL2 levels. This led to a markedly higher macrophage-to-T cell ratio in the lungs of coinfected mice, further supporting the notion that MCMV coinfection shifts local immune cell composition. Whether the elevated macrophage levels are a cause or consequence of the reduced T and B cell abundance remains to be determined.

In humans, HCMV is typically thought to have mainly negative effects on immune status and overall health, with seropositivity linked to higher mortality, cardiovascular disease, accelerated immune aging, reduced vaccine efficacy, and increased vulnerability to infectious diseases [47]. However, growing evidence indicates that the impact of HCMV on health and disease may vary throughout the lifespan [48–52]. Such findings, along with our results, imply that latent CMV infections may influence immune dynamics, in ways that benefit the host during both viral and bacterial challenges, including *Mtb*.

CMV has been shown to impair key macrophage functions like phagocytosis and TNF production [32, 34, 35], but we could not confirm this in our experiments. In contrast, MCMV-infected BMDMs efficiently internalized *Mtb*, showing no significant difference compared to naïve BMDMs. Moreover, MCMV infection enhanced macrophage control of *Mtb*, with bacterial loads increasing in the absence of MCMV but remaining stable in virus-infected cells. This could be due to MCMV-induced macrophage activation, as indicated by early TNF production, suggesting an amplified inflammatory response that may prime macrophages for a stronger antimicrobial defence against *Mtb*. The discrepancy between our in vivo and in vitro observations on the impact of MCMV on bacterial load may be attributed to the very low viral load and latent state of MCMV in vivo. In contrast, in vitro, acute infection might enable direct activation of the coinfected macrophages. Additionally, the number of coinfected macrophages in vivo might be too low to produce a similar effect.

Interestingly, introducing MCMV during an already established chronic *Mtb* infection enhanced disease progression, despite no significant differences in bacterial or viral loads. This highlights the critical role of infection timing and sequence in disease progression. Our data support the hypothesis by Cobelens and colleagues that immunologically active CMV infections - whether primary, reactivation, or reinfection - can precipitate the progression of latent TB to TB disease [7]. Our findings underscore that it may not be the mere presence of CMV, but rather the acute phase of infection that drives disease severity during coinfection. Our study did not suggest reactivation of latent MCMV due to *Mtb* coinfection. However, this may differ in a human population that is consistently exposed to various potential triggers, which could drive HCMV reactivation or even reinfection.

## Conclusions

In conclusion, our study provides novel evidence that latent MCMV infection improves disease outcome in mice following *Mtb* infection, suggesting a potential role for viral latency in modulating disease tolerance and offering new perspectives on how latent viruses may influence host resilience to heterologous infections. In contrast, acute MCMV infection seems to exacerbate disease progression. These findings emphasize the importance of considering the phase of viral infection when interpreting immune dynamics during coinfection. Further research is needed to explore the molecular mechanisms by which different phases of CMV infection influence the progression to active TB disease, with the aim of providing new insights that could inform future strategies for disease management.

## Supplementary Information

Below is the link to the electronic supplementary material.

Supplementary Material 1

## Data Availability

The datasets used and/or analyzed during the current study are available from the corresponding author on reasonable request.

## Abbreviations

TB: Tuberculosis
Mtb: Mycobacterium tuberculosis
CMV: Cytomegalovidus
HCMV: Human cytomegalovirus
MCMV: Murine cytomegalovirus
WT: Wild type
MOI: Multiplicity of infection
BMDM: Bone marrow-derived macrophages
IF: Immunofluorescence
mIF: Multiplex IF
H&E: Hematoxylin & Eosin
TBST: Tris-buffered saline with Tween20
WSI: Whole-slide imaging
