# Supplementary material for: The impact of cytomegalovirus coinfection on tuberculosis in the mouse model
Source: BMC Infect Dis. 2026 Mar 4;26:739. doi: 10.1186/s12879-026-12753-5 (PMC13063929; doi:10.1186/s12879-026-12753-5)
Supplement: Supplementary file 1 — Supplementary Material 1 [file 12879_2026_12753_MOESM1_ESM.docx]

| **Cycle No** | **1** | | | **2** | | | | **3** | | | **4** | | | **5** | | | | **6** | | | | | | | | | |  | | |
| --- | --- | --- | --- | --- | --- | --- | --- | --- | --- | --- | --- | --- | --- | --- | --- | --- | --- | --- | --- | --- | --- | --- | --- | --- | --- | --- | --- | --- | --- | --- |
| **Antibody name** | **mpt64** | | | **empty** | | | | **CD68** | | | **CD3** | | | **empty** | | | | **CD19** | | | | | | | | | | **DAPI** | | |
| **Host, Clone** | **Rabbit, polyclonal** | | |  | | | | **Rabbit, polyclonal** | | | **Rabbit,**  **SP7** | | |  | | | | **Rabbit, EPR23174-**  **145** | | | | | | | | | |  | | |
| **Supplier/Cat.#** | **Cusabio,**  CSB-PA 14949A0Rb | | |  | | | | **Abcam,** ab125212 | | | **Zytomed Systems,** RBK024 | | |  | | | | **Abcam,** ab245235 | | | | | | | | | |  | | |
| time [min] @ microwave power [Watt] | **pH6;**1min @1000,  10 min@ 100 | | | **pH6;**1min @1000,  10 min@ 100 | | | | **pH6;**1min @1000,  10 min@ 100 | | | **pH6;**1min @1000,  10 min@ 100 | | | **pH6;**1min @1000,  10 min@ 100 | | | | **pH6;**1min @1000,  10 min@ 100 | | | | | | **pH6;**  1min @1000,  **5 min**@ 100 | | | | - | | |
| 3% H_2_O_2_ Block | **10 min.** | | |  | | | | 0 | | | **0** | | |  | | | | **0** | | | | | | **0** | | | | **0** | | |
| 1x TBST washing  [min.] | 2 | 2 | 2 |  |  |  | | 2 | 2 | 2 | 2 | 2 | 2 |  |  | |  | 2 | 2 | | | 2 | | | 2 | 2 | 2 | 2 | 2 | 2 |
| Blocking | 10 min. | | |  | | | | 10 min. | | | 10 min. | | |  | | | | 10 min. | | | | | | OPAL 780 | | | | Spectral DAPI | | |
| 1x TBST washing  [min.] | 2 | 2 | 2 |  |  |  | | 2 | 2 | 2 | 2 | 2 | 2 |  |  | |  | 2 | 2 | | 2 | | | |  | | |  | | |
| Dilution | 1-200 | | |  | | | | 1-250 | | | 1-200 | | |  | | | | 1-200 | | | | | | **1-25** w. Blocking Solution | | | | 3 drops in 1000µl PBS | | |
| time [min] | 45 min. | | |  | | | | 45 min. | | | 45 min. | | |  | | | | 45 min. | | | | | | 60 min. | | | | 5 min. | | |
| Temperature | RT | | |  | | | | RT | | | RT | | |  | | | | RT | | | | | | RT | | | | RT | | |
| 1x TBST washing  [min.] | 3 | 3 | 3 |  |  | |  | 3 | 3 | 3 | 3 | 3 | 3 |  | |  |  | 3 | 3 | | | | 3 | | 3 | 3 | 3 | 3 | 3 | 3 |
| Opal HRP Polymer Rb [min.]@RT | 1-4 diluted; 10 min. | | |  | | | | 1-4 diluted; 10 min. | | | 1-3 diluted; 10 min. | | |  | | | | 1-4 diluted; 10 min. | | | | | | Wash buffer | | | | dH_2_O rinsing | | |
| 1x TBST washing  [min.] | 3 | 3 | 3 |  |  | |  | 3 | 3 | 3 | 3 | 3 | 3 |  | |  |  | 3 | 3 | 3 | | | | | 3 | 3 | 3 |  | | |
| **OPAL fluorophore**  **1:150 dilution** | 690 | | |  | | | | 520 | | | 570 | | |  | | | | OPAL TSA-DIG 1:100 in amplification buffer | | | | | | Wash buffer | | | | Mount with Prolong Gold | | |
| time [min] @ RT | 10 min. | | |  | | | | 10 min. | | | 10 min. | | | 10 min. | | | | 10 min. | | | | | | 10 min. | | | |  |  |  |
| 1x TBST washing  [min.] | 2 | 2 | 2 |  |  | |  | 2 | 2 | 2 | 2 | 2 | 2 | 2 | | 2 | 2 | 2 | 2 | 2 | | | | | 2 | 2 | 2 |  | | |
